# Editorial

**Published:** 1904-02-15

**Authors:** 


### EDITORIAL.

What shall we say about porcelain inlay filling? A recent editorial in an esteemed contemporary expresses the sentiment of some that too much time was given to papers and clinics on this subject at the National meetings last summer. This raises the question as to the place this filling material is to occupy in practice in the future. Some claim that it is only a fad and will pass away in time with other impracticable ideals; some that it is too expensive when properly done to ever become popular; and others still that it is tedious to do and will not stand the test of reasonable permanence. On the other hand, its warmest advocates are those who are able to accomplish highly artistic results from the cosmetic standpoint, and who are most strenuous in claiming for it a permanence not excelled, if equaled, by any other filling material. That it has a value from its cosmetic, as well as its compatible nature, can not be questioned. It remains to be demonstrated that it is less conducive to recurrent caries, and that it strengthens frail teeth rather than taxes their integrity under the stress of mastication. The fact is however patent that there is a growing interest in this work by the profession generally. This is shown by the literature, the demand for facilities in the way of materials and appliances for constructing it, and the demand for instruction in the colleges. The University of Michigan recently offered a course to its alumni and limited the number to such as could be accommodated. The result was that in less than ten days more than twice as many applied for the course than could be accommodated. The character of the men who sought the opportunity were such as to indicate also that the better class of operators believe that this work has a value and place not now given to it. Its advocates are rational and we must weigh its claims carefully.

